# A Study on the Sustainable Development of Water, Energy, and Food in China

**DOI:** 10.3390/ijerph16193688

**Published:** 2019-09-30

**Authors:** Lei Jin, Yuanhua Chang, Xianwei Ju, Fei Xu

**Affiliations:** 1 School of Economics and Management, China University of Petroleum, Beijing 102249, China; jinlei@cup.edu.cn (L.J.); 2017217028@student.cup.edu.cn (X.J.); 2 Department of Mathematics, Wilfrid Laurier University, Waterloo, ON N2L 3C5, Canada; fxu@wlu.ca

**Keywords:** water, energy, food, sustainable development, system dynamics

## Abstract

It is of great significance to deal with the relationship between external factors and the water-energy-food internal system for China’s sustainable development. This paper takes China as the research object, uses the system dynamics method to construct a model for China’s water-energy-food system, and introduces the “two-child” policy and trade friction as the scenario parameters for simulation. The main results of scenario simulation can be summarized as the following three points. In terms of water, the trade friction will hinder China’s industrial water consumption into a low-consumption stage. In terms of energy, both the trade friction and the “two-child” policy, will not change the increasing trend of energy demand. In terms of food, if there is strong response to the “two-child” policy, there will be insufficient food inventory under the current capacity and import ratio. In short, this paper takes the sustainable development of water-energy-food as a starting point and puts forward policy suggestions on the comprehensive formulation of policies.

## 1. Introduction

As we all know, resources are not inexhaustible. China, as a populous country, faces enormous pressures on water, energy, and food. For water resources, the per capita water consumption in China is only about 1/4 of the world’s average level. As such, China has been listed as one of the 13 water-deficient countries in the world. In terms of energy, in 2018, the external dependence on oil of China was approaching 70%. Hence, China has significant energy security issues. In terms of food, the Development Research Center of the State Council expects that the total food demand in China will peak in 2030, and the food supply problem is becoming increasingly severe. This paper aims at analyzing how China’s water-energy-food system can meet social needs and achieve sustainable development under limited resources.

In the late 1980s, it was noted that water security and food security were very closely related [1]. It was not until 2011 that the water-energy-food bond relationship was first proposed at the Bonn conference. It is believed that such relationship can be examined by taking into account water, energy, food, and other factors, which has attracted the attention from researchers and beyond. The production of energy consumes water resources. The sewage discharged from energy consumption further affects the quality of water resources. The production of food needs irrigation, and thus water pollution directly affects food production. As strategic resources for the survival and development of humans, water, energy and food affect the stability and security of the society. In this paper, we examine the water-energy-food system and the supply and demand variables including imports, production, and consumption. Water, energy, and food resources are mutually constrained, and jointly affect China’s sustainable development.

In particular, we take into account the supply and demand variables such as imports, production, and consumption, and build a system dynamics model for China’s water-food-energy system. Considering China’s current political and economic environment, the trade frictions and the “two-child” policy will be incorporated into the scenario simulation. Our study will explore the impact of trade friction and “two-child” policy on China’s water-food-energy system. The study aims to provide useful insight into the future sustainable development.

The rest of this paper is organized as follows. Section 2 presents a literature review. Section 3 describes the characteristics of water-energy-food resources in China. Section 4 establishes the system dynamics model, including drawing a causality diagram, flow diagram, setting parametric equations, and model checking. Section 5 presents a simulation, prediction, and scenario analysis. The last Section provides conclusions and suggestions

## 2. Literature Review

In the past decade, many scholars have researched the sustainable development of resources such as water, energy, and food. This research stream is driven by three factors, including the reduction of resources, the supply crisis of resources, and the deficiency of management strategies [2]. Generally speaking, most research in the literature focuses on either a single resource or a pair of related resources. Traditionally, research, especially quantitative studies, on the relationship between three resources is relatively rare. Since 2011, there have been an increasing number of studies on the water-energy-food system, as it has become a major concern in the sustainable development of many countries.

In 2005, J. Diamond realized that it is essential for human society to try to solve some complicated and mutually influential problems [3]. Mohammad and Nadir (2017) believed that these problems, including water-energy-food linkages, would be the most basic threat to the survival of humans [4]. Bazilian et al. proposed the CLEW (Climate-Land-Energy-Water) model in 2011, and constructed a theoretical analytical framework from four areas: climate, land, energy, and water [5]. Some scholars adopt the Nexus approach, an integrated management method, to emphasize cross-sectoral integrated management of natural resources such as water, energy, land, and biomass. Such integrated approaches will help improve water, energy, and food security, environmental and climate security, and eventually also political security [1,6]. Similarly, in developing countries, the emergence of many problems is related to the production, sale, and use of water, energy, and food. Scholars in China started such research relatively late. Most of their studies focused on three major fields including energy, economy, and environment (3E system) [7,8,9,10,11], or on single areas (water, energy, or food). The interaction in the water-energy-food system had been rarely studied [12].

Some scholars take water resources as the main line of the research, and consider less about economic factors. Bridge et al. (2013) conducted research on the relationship between water and energy in extreme dry weather conditions in Texas, USA [13]. Cai et al. (2018) studied the water-energy-food system with a focus on the water resource and proposed a comprehensive management strategy for the system [14]. Vandone et al. (2018) used a multifactor market model to investigate how the water industry stock prices react to changes in agriculture and energy prices [15]. With the increased awareness to the water-energy-food system, the research on the correlation between water, energy, and food has attracted more and more attention. Zhang et al. (2014) studied the ties in the water-energy-food system from the perspective of industry (tourism, etc.) [16]. Li et al. (2017), on the other hand, investigated the inner links in such system from the perspective of energy supply [17]. In addition, the effects of population growth, technological advancement, environmental protection, and policy on the evolution of water-energy-food systems had been considered in the literature [18,19]. The water-energy-food systems can also be studied from the national level to the regional and urban levels [20,21,22].

Qualitative research were carried to obtain policy recommendations [2,23,24]. On the other hand, quantitative research was developed to simulate the sustainable development of the water-energy-food system [25,26,27,28]. Sahin et al. (2014) used case studies to investigate interactions in a water-energy-food system [29]. Scholars have applied the DEA (Data Envelopment Analysis) model to the evaluation of the input and output efficiency of water, energy, and food in different regions of China [17]. Zhang et al. (2017) conducted a global sensitivity analysis to quantify parameters in water, energy, food, and the environment [30]. Xu et al. (2019) used the coupling and coordination degrees to measure the core water-energy-food nexus in China [31]. Fan (2014) employed an ARIMA (Autoregressive Integrated Moving Average) model to predict the evolution of various resources [32]. Xie and Li (2009), on the other hand, developed a genetic algorithm to forecast various resources in the future [33]. Moreover, some scholars used the System Dynamics (SD) model to analyze the relationship between water-energy-food in Beijing and other places [18,22].

Recent studies on water-energy-food systems indicated that different countries have different research methods and research perspectives on the water-energy-food system. There exist complex connections between water, energy, and food, and such relationship can be influenced by many factors including society and climate. It is essential to keep the balance in the water-energy-food system to maintain sustainable development of society. In order to formulate appropriate policies, the sustainable development of water, energy, and food resources should be taken in to account.

## 3. The Characteristics of Water-Energy-Food Resources in China

In terms of current national conditions of China, China is a country suffering from drought and water shortage. The water resources in China are not abundant and distributed unevenly among regions. With the increase of population, the per capita water resources in many areas of China are lower than the international standard. China’s water resources are mainly consumed in industry, life, agriculture, and ecology. In terms of the proportion of water consumption, agricultural and industrial water has relatively high consumption proportion. The total amount of consumed industrial water has declined in recent years. With the improvement in living standard and awareness of environmental protection, the total amount of domestic water and ecological water consumption maintained continuous high growth rates at 4.10% and 2.38%, respectively.

Meanwhile, China is the world’s second largest energy consumption country with abundant coal resources and less gas and oil. Secondly, a large amount of waste gas is produced in the process of energy consumption, which should be considered. As the proportion of coal and oil consumption in non-clean energy is far higher than that of other energy sources, in this paper, we use sum of coal and oil consumption to represent the total consumption of non-clean energy. The energy consumption structure of non-clean energy has been fluctuating between 83% and 93%. As a result, the amount of waste gas and water produced in the process of energy consumption is increasing. However, the growth rate of wastewater discharge has slowed down in recent years.

As an important material for the survival of human, food is indispensable. The food and agriculture organization of the United Nations defines foods as cereal, mainly including wheat, beans, coarse foods, and rice. As a populous country, China is increasingly dependent on food. With the increase of population, the consumption of food is also increasing gradually. In 2016, food consumption reached 657.303 million tons, and the demand for food will continue to increase in the future. Food is used in industrial production, for human consumption, to feed live inventory, and as seeds. With the improvement of people’s living standard, the demand for meat products increased gradually, which could lead to the increase in the consumption of feed food. With the development of industry, food consumed in industry has gradually increased. The increase in food demand will challenge the safety balance between food production and food import in China in the future.

## 4. Modeling the Water-Energy-Food System in China and Testing Its Effectiveness

The cyclic process of system dynamics is a specific dynamic simulation process set designed for the research direction. Thus, setting system boundaries plays a crucial role in the accuracy of the system and the definition of research content. Based on the timeliness of the research and the availability of the data, we chose data from the last 20 years (1997–2016) as the research basis. We explored the restriction and interaction within the system, and predicted the resource demand for the next 10 years (2017–2026) for different development scenarios. However, we have not yet obtained the data for 2017 and 2018. When the 2017–2018 data are available, we plan to verify the model prediction results.

### 4.1. Model Design

#### 4.1.1. Causality Diagram of China’s Water-Food-Energy System

The most important step in establishing system dynamics is to determine the causal diagram. Through continuous analysis and debugging, we integrated GDP (Gross Domestic Product), population, and environment into the water-energy-food system in China to develop a complex circulatory system. In order to present intuitively, the system is divided into three subsystems: water, food, and energy.

**Figure 1:**
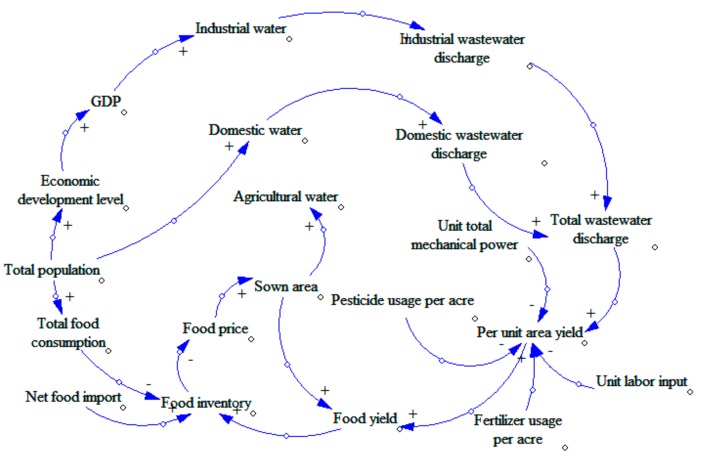
Causality diagram of China’s water subsystem.

**Figure 2:**
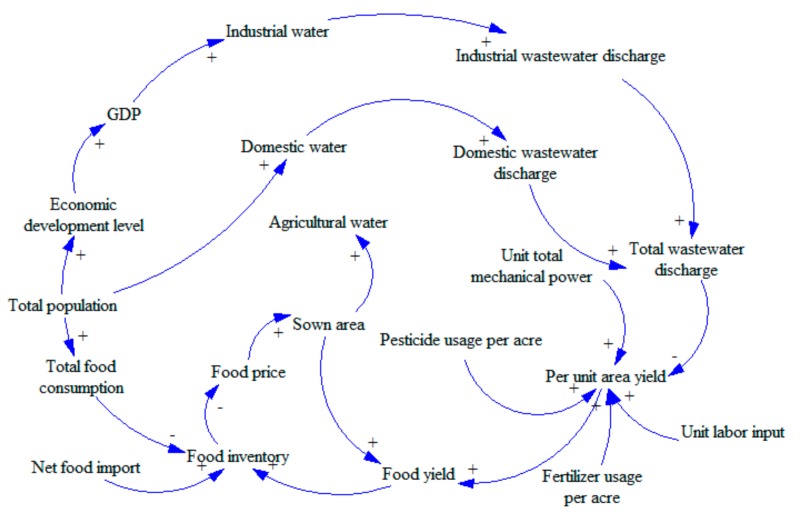
Causality diagram of China’s food subsystem.

**Figure 3:**
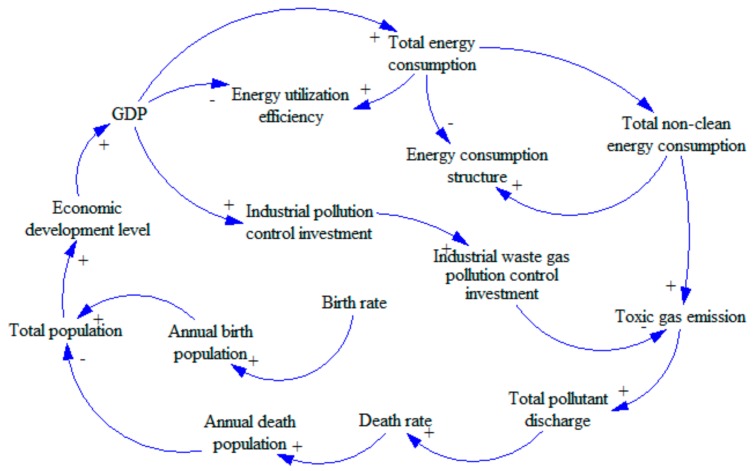
Causality diagram of China’s energy subsystem.

(1) Water subsystem (see Figure 1). We will study the water subsystem from the perspective of water consumption. We will consider industrial water, agricultural water, domestic water, and ecological water. These factors also have a circular feedback relationship with changes in population and energy consumption. There are three loops in Figure 1.
  Loop 1: GDP—Industrial pollution control investment—Industrial wastewater pollution control investment—Industrial wastewater discharge—Total wastewater discharge—Death rate—Annual death population—Total population—Economic development level—GDP.
  Loop 1 is a positive feedback loop. With the increase in GDP, the industrial pollution control investment will be increased as well as the industrial wastewater pollution control investment. As a result, the wastewater processing capacity will be increased, and the industrial wastewater discharge will be decreased. With less pollution, the environment will be friendlier, and the death rate will decline. The economic development level will gradually increase, leading to an increase in GDP, which forms a positive feedback loop.
  Loop 2: Total population—Domestic water—Domestic wastewater discharge—Total wastewater discharge—Total pollutant discharge—Death rate—Annual death population—Total population.
  Loop 2 is a negative feedback loop. With the increase in total population size, the consumption of domestic water will increase, leading to an increase in wastewater emission. The increase in wastewater emission will cause increased pollution, leading to a higher death rate. As such, the total population size will decrease, implying that it is a negative feedback loop.
  Loop 3: Total population—Economic development level—GDP—Industrial water—Industrial wastewater discharge—Total wastewater discharge—Total pollutant discharge—Death rate—Annual death population—Total population.
  Loop 3 is a negative feedback loop. The increase in total population will raise the economic development level, causing increased GDP. The increase of GDP will lead to the development of industry, causing increases in the consumption of industrial water. Thus, there will be more wastewater emissions, causing environmental degradation. The deteriorated environment will then affect the health of human beings, leading to a decrease in the total population size, which forms a negative feedback loop.
(2) Food subsystem (see Figure 2). The subsystem of food resources will be studied from the perspective of food inventory. We will explore the cyclical feedback relationship between variables including food consumption, food production, net food imports and food inventory, and the changes in the population and pollution emissions.
  Loop: Food yield—Food inventory—Food price—Sown area—Food yield.
  This loop is a negative feedback loop. With the increases in food yield, the food inventory increases, which will lead to the decline of food prices. When the market price drops, farmers will reduce the sown area, leading to decreased food yield, which forms a negative feedback loop.
(3) Energy subsystem (see Figure 3). The energy resource subsystem will be studied from the perspective of energy consumption structure. We will explore the circular feedback relationship between variables including consumption of non-clean energy, investment in the control of waste gas pollution and emission of pollutants, and the total energy consumption and level of economic development.
  Loop: Economic development level—GDP—Total energy consumption—Total non-clean energy consumption—Toxic gas emission—Total pollutant discharge—Death rate—Annual death population—Total population—Economic development level.
  This loop is a negative feedback loop. Increasing economic development level will lead to increased GDP. The increase in GDP will lead to an increase in total energy consumption. As the main type of energy, more non-clean energy will then be consumed, leading to an increase in toxic gas emission. The polluted air will cause higher death rate, leading to a decrease in total population. Less population will slow economic growth [34], which forms a negative feedback loop.

#### 4.1.2. Stock-Flow Diagram of China’s Water-Food-Energy System

**Figure 4:**
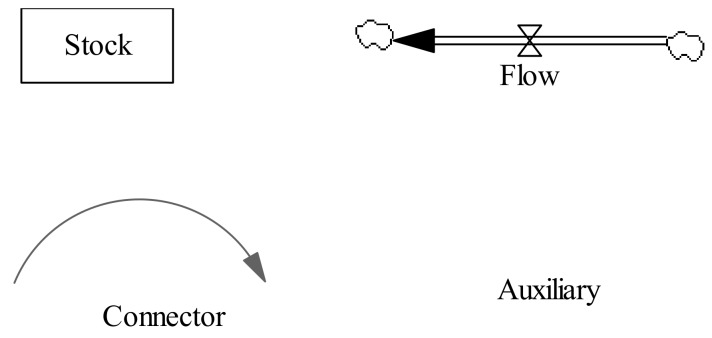
Building blocks of system dynamics models.

**Figure 5:**
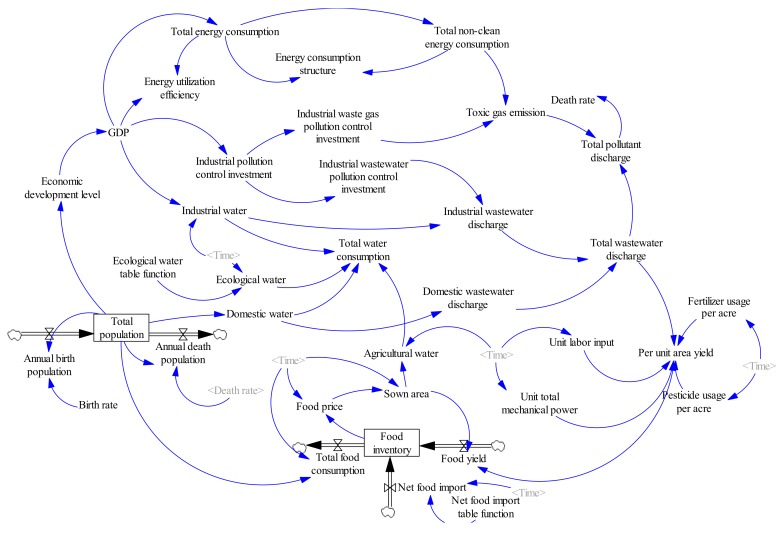
Stock-flow chart of China’s water-energy-food system.

The basic building blocks for a flow diagram of system dynamics models are stocks flows, connectors, and auxiliaries [35] (Figure 4). Stocks represent accumulations. Examples of stocks are total population (TP) and food inventory (FI). Stocks represent the ‘traces’ left by an activity. Material in a stock exists at a given point in time and persists even when activities end. Flows represent activities or actions in a stock that transport quantities into or out of a stock instantaneously or over time. Examples of flows are annual birth population (ABP), annual death population (ADP), total food consumption (TFC), food yield (FY), and net food import (NFI). Mathematically, the relationship between stocks and flows can be described using the following integral form [36,37].

(1) $$S\mathrm{tock}(t)=\overset{t}{\underset{t_{0}}{\int }}\left[Inflow(s)-Outflow(s)\right]ds+Stock(t_{0}),$$

where *t*_0_ is the initial time, *t* is the current time, *Stock*(*t*_0_) is the initial value of the stock, *Inflow*(*s*) and *Outflow*(*s*) are flow rates into and out of a stock at any time *s* between the initial time *t*_0_ and current time *t*. *Inflow*(*s*) and *Outflow*(*s*) have the units of *Stock*(*t*) divided by time. Connectors (arrows shown in Figure 4) establish relationships between various elements of the model and move information as inputs for decisions or actions. Auxiliaries house graphical and built-in functions (no shape in Figure 4). Examples of auxiliaries are energy utilization efficiency (EUE), energy consumption structure (ECS), total pollutant emission (TPE), etc. The stock-flow diagram for the model is shown in Figure 5.

### 4.2. Initial Parameters and Equations of the Water-Energy-Food System in China

The software Vensim and Stella are widely adopted by system dynamics models for simulation applications [38,39]. In this study, the software Vensim was employed. We considered 35 variables related to water, energy and food in China from 1997 to 2016 (20 years). The data mainly come from the China’s Statistics Bureau, the statistical database of China’s economic and social development, the national food and oil information center, and the statistical yearbook of China. For some variables, due to missing data for a few years, we used SPSS software to create supplementary data. For the energy consumption structure data of non-clean energy, we measured it using the ratio of oil and coal to total energy consumption. Energy utilization efficiency is measured by the amount of energy used per unit of GDP. The economic development level is measured by per capita GDP. The discharge of wastewater is obtained by taking sum of industrial wastewater discharge and domestic wastewater discharge. The unit labor force is characterized by the employment of the primary industry per unit of sown area. The birth rate is averaged at approximately 12.7725‰.

The best regression equations were selected according to two criteria: the equation had to be logical (i.e., it had to provide a causal explanation for an observed trend), and it needed to have a high goodness of fit (i.e., R^2^) [38,40]. The main variables and the final regression equations used in this model are described in Appendix A.

### 4.3. Model Test

In order to ensure the robustness and rationality of the system, sensitivity test and error analysis of the water-energy-food system were carried out. If there is no significant change in the behavior of the system with some perturbation in some system parameters or slight change in equation structure, then the structure of the model is overall reasonable, and the model is relatively robust. Sensitivity test variable is birth rate. Target variables include total food consumption, total energy consumption, total pollutant discharge, total water consumption, energy efficiency, GDP, and economic development level. The range of birth rates in the past 10 years is roughly between ± 4%. Therefore, we set the birth rate to change by ± 4%.

Sensitivity test showed that the birth rate fluctuates between ± 4%, and most of the target variables vary within 6%. Due to the close relationship between individual variables, the most sensitive fluctuation is close to 15%, which is consistent with the fact. The error analysis of the model is mainly to verify whether the results of the model fitting meet the historical actual values. We carried out error analysis of the target variables in the system. The errors of population, GDP, energy consumption structure, total water consumption, total food consumption, and economic development level are all within 10%, showing a good degree of fit.

## 5. Simulation, Prediction, and Scenario Analysis

### 5.1. Simulation and Prediction

**Figure 6:**
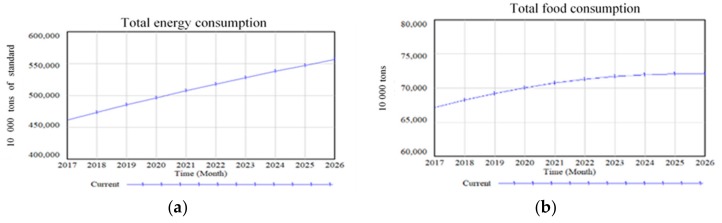

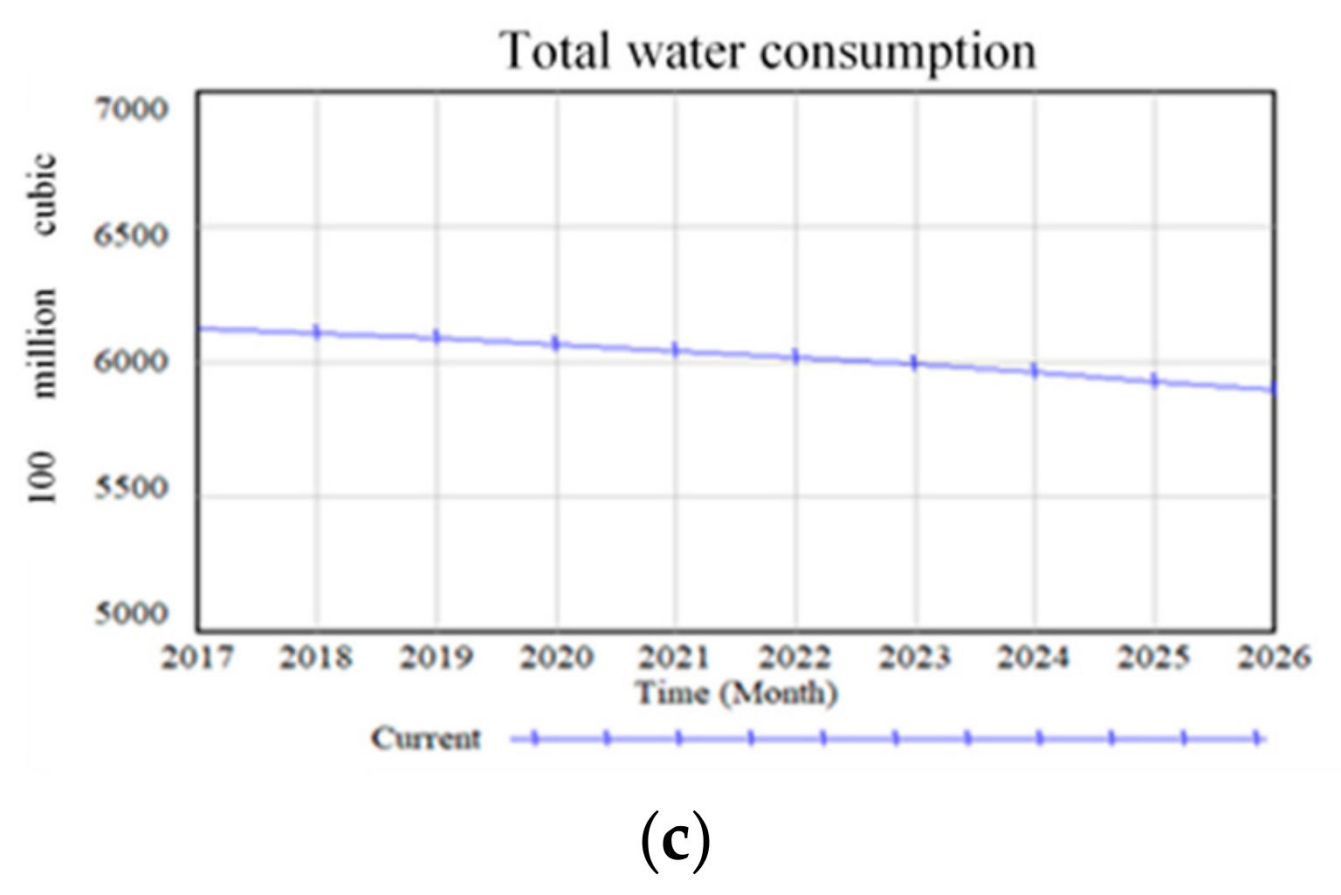
Predictive simulation results.

According to the simulation results of the water-energy-food system model, considering the current development level, we obtained the forecast results of China’s resource demand from 2017 to 2026, as shown in Figure 6.

As is shown in Figure 5, in the future, the total energy consumption will gradually increase, the total food consumption in China will increase but with slower speed, and total water consumption shows a small downward trend. We conjecture that the reduction in total water consumption is related to China’s proposed “Made in China 2025” strategic goal. “Made in China 2025” requires an improvement in the quality and level of manufacturing, which will inevitably lead to the optimization of industrial structure in the next 10 years. With upgraded industrial structure, steel and other processing and smelting manufacturing industries will shrink. In recent years, the proportion of investment in processing and smelting manufacturing industries fell by nearly 10%. On the other hand, the investment in high-tech industry is gradually increasing, leading to a gradual reduction in industrial water consumption in the future. However, China’s total water consumption in 2026 only decreased by 25.557 billion cubic meters compared with that in 2016, which is not significant. In general, China still faces serious resource constraints in energy, food, and water.

### 5.2. Scenario Analysis

The water-food-energy system will be influenced by many factors such as policy, economy, population, foreign trade, and their changes in which a country is located. It can be seen from earlier analysis that China’s water-food-energy still faces challenges in the future. Combining China’s current political and economic environment, we selected the trade friction between China and the United States and the “two-child” policy as the parameters of the scenario simulation. We analyzed the water-food-energy system under those changes in the external political and economic environment (Table 1).

The 13th five-year plan for 2015 set new targets and requirements. The plan proposed that, by 2020, the total population of the country will be around 1.42 billion people. Then, China will be ranked as a high-income country. That is to say, according to the criteria set by the World Bank, China’s per capita income will reach 12,056 US dollars. In terms of economic objectives, the economic growth rate of China has slowed down significantly due to the trade friction between China and the United States. Ren Zeping, the president of the Evergrande Group Economic Research Institute, believed that China’s “increasing speed shift” in 2019 will enter the “economic L-type” bottoming period, and the economic downturn will be large. In terms of the population target, China implemented the “two-child” policy in 2015, which means that a couple can have two children. The “two-child” policy ended the “one-child” policy that had been in place for 35 years. China’s population in 2018 was nearly 1.4 billion, accounting for 18% of the world’s total population. If the implementation of the “two-child” policy leads to a surge in population, it will pose a serious challenge to China’s water-energy-food system. However, although the birth rate increased significantly in 2016 and 2017, it was followed by a decline in 2018 (reduced by about 2 million from the previous year). In summary, this study intends to simulate the impact of trade frictions on China’s water-food-energy system by changing the parameters of economic development level, and to simulate the impact of the “two-child” policy on China’s water-food-energy system by changing the birth rate parameters.

In the following, from the perspective of indicators, the changes of variables in the five scenarios were compared with the results in the base period. We found that the indexes are quite different in different scenarios. The observation indexes include total energy consumption, energy consumption structure, energy utilization efficiency, total water resource consumption, industrial water consumption, agricultural water and food consumption, and food inventory.

#### 5.2.1. Analysis Results of Water Resource Subsystem Scenario

**Figure 7:**
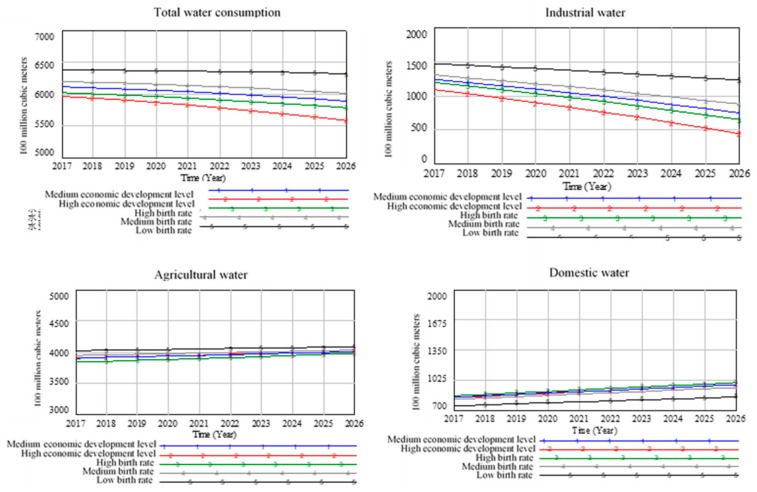
Scenario prediction curve of water subsystem.

As shown in Figure 7, when the birth rate is low, i.e., the population target of the 13th Five-Year Plan is not reached, the total amount of water consumption will not decrease, mainly caused by the increase in industrial water consumption. There is no significant change in agricultural water consumption. Although about 65% of water consumption is used for agriculture, since sown area is fixed, there should not be much change in the amount of irrigation water. With the decrease of birth rate, there is reduction in domestic water consumption.

If China enter the ranks of high-income countries, the total amount of water consumption will decrease. When per capita GDP reaches the standards of developed countries, China’s industrial structure will be optimized, and thus industrial water consumption will be reduced. The consumption of industrial water will enter a low-cost stage without reducing economic development capacity. Thereby wastewater discharge will be reduced. If economic growth rate of China is affected by the trade friction between USA and China, the total amount of water consumption under medium economic development level will be higher than the that under the high economic development level. The trade friction will hinder China from entering the stage of low consumption of industrial water. Therefore, it is essential for China to strictly control industrial water consumption from the source and optimize the industrial structure. Meanwhile, since agricultural water use is still the mainstay of total water consumption, increasing investment in agricultural water-saving irrigation technology and improving agricultural irrigation technology capacity are inevitable in achieving sustainable consumption of water resources.

#### 5.2.2. Analysis Results of Energy Subsystem Scenario

**Figure 8:**
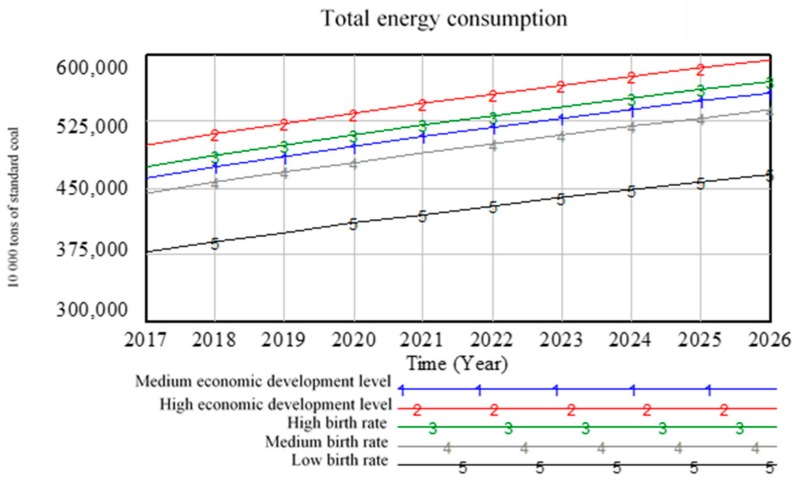
Scenario prediction curve for total energy consumption.

The total energy consumption is greatly affected by changes in population and changes in economic development levels as shown in Figure 8.

Under high economic development level, the total energy consumption is the high. The energy consumption is low when the birth rate is low. Regardless of the changes in economic development level and birth rate, the trend of energy demand is rising, which means that neither trade friction nor “two-child” policy cannot change the upward trend of energy demand. In this case, the supply and demand of energy is a big issue. Nearly 87% of China’s energy consumption comes from coal and oil, which puts tremendous pressure on China’s national energy security, because in the future, with the increase in energy demand, China can only import energy or develop new energy sources as non-renewable energy is limited. In order to achieve sustainable development, promoting the diversification of energy consumption structure is the fundamental solution to the energy security problems.

#### 5.2.3. Scenario Analysis Results of Food Subsystem

**Figure 9:**
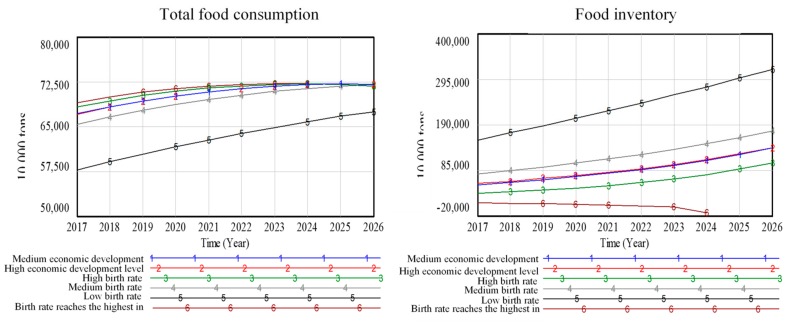
Scenario prediction curve of food subsystem.

The amount of consumed food is mainly affected by the number of people and as such is not sensitive to the changes in the level of economic development. There exists a limit in the sensitivity of food consumption to the population, which constrains the population growth. Here, we set the sixth scenario, in which the birth rate is 13.22‰. Note that the birth rate 13.22‰ exceeds the historical average birth rate. We examined the behaviors of the model when the population growth rate exceeds the expected population growth rate of China (see Figure 9).

As shown in Figure 9, when the birth rate is 10.94‰ (line 5) i.e., the size of the newly born population decreases, the food consumption also decreases, displaying a positive relationship. With the decrease in food consumption, As shown in Figure 6, when the birth rate is 10.94‰ (line 5) i.e., the size of the newly born population decreases, the food consumption also decreases, displaying a positive relationship. With the decrease in food consumption, correspondingly, the food inventory will increase, which shows that food consumption is mainly related to population size. Excluding the low birth rate scenario, the total food consumption in other scenarios will gradually converge to 725 million tons by 2026, which implies that there is a ceiling in food production due to the limited cultivated land area in China. In the absence of substantial breakthroughs in agricultural technology, such ceiling in food production will persist. When the birth rate is 13.22‰ (line 6), the prediction results show that there will be insufficient food inventory in 2024. Such results indicate that if the effect of the comprehensive implementation of the “two-child” policy in the future is significant, which leads to a birth rate higher than the historical average, there will be shortage in the food inventory when the production capacity and import ratio remain unchanged. The above conclusion is consistent with the conclusion of Wang et al. (2009) on the study of food production in China [18]. In order to maintain a basic balance between the supply and demand of food in china, it is essential to ensure a certain amount of investment in agricultural technology, and effective implementation of macro policies. Otherwise, there will be shortage of food supply [18].

Although the birth rate decreased in the past two years, if the effect of the second child policy is significant, the birth rate may still reach 13.22%. Under the current production capacity and import rates, the food inventory will run out, which will bring new problems to the food supply in China. In order to meet the food demand, China can either increase the import or increase the food production. However, increasing food import will seriously threaten the food security of the country. For national security, the state requires that the rate of food self-sufficiency should be maintained at no less than 90%. By managing the implementation intensity of the “two-child” policy, and increasing the yield of food per unit area to ensure the security of food supply, we can realize the co-prosperity and coexistence of human beings and resources to achieve sustainable development.

## 6. Conclusions

This paper conducted simulation and scenario analysis for a water-energy-food model in the next 10 years for China. The following main conclusions were obtained. From the perspective of sustainable development of water resources, the future reduction in the consumption of water resources in China will mainly be due to the technological innovation and the upgrade of industrial structure. However, the Sino-U.S. trade friction can prevent China’s industrial water consumption from entering a low-consumption stage. From the perspective of sustainable energy development, whether there is increase in the economic development level or a population decrease, the trend of energy demand is constantly rising. The contradiction between energy supply and demand will become more and more serious. From the perspective of sustainable development of food, there is an upper limit on total food consumption due to the limited arable land. If there are strong responses to the “two-child” policy, under the current production capacity and import ratio, there will be insufficient food inventory. Based on this research, we put forward the following policy suggestions.

(1) Water resources

In order to realize the sustainable development of water resources, it is necessary to reduce the total consumption of industrial water and agricultural water. In terms of industrial water, enterprises can increase the utilization efficiency of water resources through technological progress, adjusting the industrial structure to compress the industries with high water consumption, and increasing the reuse of waste water. In terms of agricultural water, the government should encourage farmers to stop using traditional extensive irrigation methods and popularize water-saving agricultural water facilities. Improving the capacity of agricultural irrigation technologies has a major impact on the reduction of water consumption.

(2) Energy

It is essential to develop renewable energy, optimize energy consumption structure, promote technological innovation, and improve energy efficiency. In the future, with increased energy demand and limited reserve on non-renewable energy, in order to avoid future energy shortage and excessive dependence on import, it is necessary to develop new renewable energy. This is the only solution to the problem of energy supply and demand. Improving energy efficiency is an inevitable requirement for the sustainable development of China. Furthermore, we need to promote technological innovation, and accelerate the improvement of energy efficiency through optimizing energy consumption structure and industrial structure.

(3) Food

We need to increase the food yield per unit area, adjust the structure of food varieties, and ensure the safety of food supply. Food consumption is mainly affected by the population size. Rapid growth of population will lead to insufficient food inventory. Only by appropriately implementing the “two-child” policy can we realize the co-prosperity and sustainable development of human beings and resources. Meanwhile, there still exists the “simultaneously increase in three agricultural quantities” phenomenon in China. It is necessary to deepen agricultural reform, adjust the structure of food varieties, motivate farmers to grow low-yield crops, and reduce import dependence on low-yield crops such as US soybeans. Secondly, in order to improve food self-sufficiency, it is necessary to increase the food production capacity under the condition of limited arable land. Increasing the productivity of low-yield crops is particularly important. By reducing the import of low-yield crops, national demand for food will be met and national agricultural security will be in a self-supplying range.

With the development of society, technological progress plays an important role in resource consumption. This paper opens up a wide area of future work. In the future, we will examine the influence of various aspects of technology investment on resource consumption and pollutant emissions. Secondly, our research focuses only on China and its political and economic environment. In the future, we will study the water, energy and food in other countries and regions and develop more general models. Thirdly, the phenomenon “simultaneously increase in three quantities” is mainly related to the structure of food variety in China. Taking the food variety structure as the key variable to study, we can conduct investigations on refining the food variety structure and estimating future food import volume.

## Figures and Tables

**Table 1:** Scenario parameter setting and explanation.

Category	Scenario	Meaning	Parameter Setting
Population scenario	Low birth rate	The population target for the 13th five-year plan will not be met, and the fertility rate is low	The birth rate was set at 10.94‰ (according to the 2018 birth rate)
	Median birth rate	The population target for the 13th five-year plan will be met. The population target of 1.42 billion will be reached by 2020	The birth rate is set at 12.3‰
	High birth rate	The response to the “two-child” policy is strong, exceeding the target of the 13th five-year plan	The birth rate is set at 13‰
Economic scenario	Medium economic development level	Affected by the trade friction between China and the United States, it is impossible to step into the ranks of high-income countries by 2020	Keep the current level of economic development
	High economic development level	The economic goal of the 13th Five-Year Plan is achieved, and China enter the ranks of high-income countries in 2020	GDP per capita is increased by 30%

## Appendix A

**Table A1:** Main variables and parameters index of China’s water-food-energy system.

Notation	Variable Meaning	Variable Type	Function Type	Mathematical and Logical Relational Expressions
*IWPCI*	Industrial wastewater pollution control investment	Auxiliary variable	Linear function	*IWPCI* = − 37.907 + 0.573 307**IPCI*
*DW*	Domestic water	Auxiliary variable	Linear function	*DW* = − 1 982.63 + 0.020 282**TP*
*IWGPCI*	Industrial waste gas pollution control investment	Auxiliary variable	Linear function	*IWGPCI* = 21.066 6 + 0.283 667**IPCI*
*TNEC*	Total non-clean energy consumption	Auxiliary variable	Linear function	*TNEC* = − 1966.344 + 0.88**TEC*
*GDP*	GDP	Auxiliary variable	Linear function	*GDP* = − 11 241.1 + 1.389 8**EDL*
*DR*	Death rate	Auxiliary variable	Linear function	*DR* = 5.740 43 + 0.001 809**TPD*
*UTMP*	Unit total mechanical power	Auxiliary variable	Linear function	*UTMP* = −659.672 + 0.332 272**Time*
*FUPA*	Fertilizer usage per acre	Auxiliary variable	Linear function	*FUPA* = − 21 214.1 + 10.803**Time*
*PUPA*	Pesticide usage per acre	Auxiliary variable	Linear function	*PUPA* = − 698.659 + 0.355 335**Time*
*TFC*	Total food consumption	Rate variable	Piecewise function	*TFC* = IF THEN ELSE(*Time* ≤ 2007, − 45 323.4 + 0.734 912**TP*, − 1.708 61e + 006 + 18.361**TP* − 2.892e − 010**TP*^^3^)
*IW*	Industrial water	Auxiliary variable	Piecewise function	*IW* = IF THEN ELSE(*Time* ≤ 2010,991.106 + 0.001 851**GDP* − 1.805e − 009**GDP*^^2^,1 760.3 − 0.000 613**GDP*)
*AW*	Agricultural water	Auxiliary variable	Piecewise function	*AW* = IF THEN ELSE(*Time* ≤ 2000, 3 644.82,919.601 + 0.026 062**SA*)
*SA*	Sown area	Auxiliary variable	Piecewise function	*SA* = IF THEN ELSE(*Time* ≤ 2004, 4.035 72e + 006 − 1 962.98**Time*, 21 801.3**FP*^^0.175125^)
*ULI*	Unit labor input	Auxiliary variable	Piecewise function	*ULI* = IF THEN ELSE(*Time* ≤ 2003, 3.262, 268.065 − 0.132 077**Time*)
*FP*	Food price	Auxiliary variable	Piecewise function	*FP*= IF THEN ELSE(*Time* ≤ 2005, 3 871.46 + 0.475**FI* − 2.03e − 005**FI*^^2^, − 18 616.2 + 2 909.44**LN*(*FI*))
*IPCI*	Industrial pollution control investment	Auxiliary variable	Logarithmic function	*IPCI =* − 2 147.66 + 205.301**LN*(*GDP*)
*TEC*	Total energy consumption	Auxiliary variable	Logarithmic function	*TEC* = − 1.462 49e + 006 + 141 032**LN*(*GDP*)
*DWD*	Domestic wastewater discharge	Auxiliary variable	Exponential function	*DWD* = *EXP*(8.739 99 − 2 042.77/*DW*)
*PUAY*	Per unit area yield	Auxiliary variable	Multivariate linear function	*PUAY* = 5 649.83 − 71.401**UTMP* − 601.068**ULI* + 2.545**FUPM* − 19.47**PUPM* + 0.774**TWD*
*TGE*	Toxic gas emission	Auxiliary variable	Multivariate linear function	*TGE* = − 173 447 − 420.831**IGPCI* + 2.481**TNEC*
*IWD*	Industrial wastewater discharge	Auxiliary variable	Multivariate linear function	*IWD* = 71.006 + 0.115 316**IW* − 0.009 569**IWPCI*
*EDL*	Economic development level	Auxiliary variable	Multivariate linear function	*EDL* = 2.045 06e + 007 − 248.913**TP* + 5.492e − 009**TP*^^3^
*ECS*	Energy consumption structure	Auxiliary variable	\	*ECS* = *TNEC*/*TEC*
*EUE*	Energy utilization efficiency	Auxiliary variable	\	*EUE* = *TEC*/*GDP*
*TWC*	Total water consumption	Auxiliary variable	\	*TEC* = *IW* + *AW* + *DW* + *EW*
*TWD*	Total wastewater discharge	Auxiliary variable	\	*TWD* = *IWD* + *DWD*
*TPD*	Total pollutant discharge	Auxiliary variable	\	*TPD* = *TWD* + *TGE*
*TP*	Total population	Stock variable	\	*TP* = *INTEG*(*ABP* − *ADP*, 123626)
*ABP*	Annual birth population	Flow variable	\	*ABP* = *TP***BR*/1000
*ADP*	Annual death population	Flow variable	\	*ADP* = *TP***DR*/1000
*NFI*	Net food import	Flow variable	\	*NFI* = *Net food import table function(time)*
*EW*	Ecological water	Auxiliary variable	\	*EW* = *Ecological water table function(time)*
*FY*	Food yield	Flow variable	\	*FY* = *PUAY***SA*/10,000
*FI*	Food inventory	Stock variable	\	*FI* = *INTEG*(*FY* + *NFI* − *TFC*, 9106.45)
*BR*	Birth rate	Constant	\	12.7725

Remarks: The variable that needs to be fitted, whose function type has been reported in the table, and its mathematical expression is shown in the last column. The R^2^ (goodness of fit) of the fitting equation is above 0.9. “\” indicates that the variable does not need to be fitted, some of the logical expressions are already defined in the text, and another part of the logical expression can be reasoned by common sense.
